# When states require fully insured employers to cover in vitro fertilization (IVF), what do self-insured employers provide?

**DOI:** 10.1007/s10815-025-03756-3

**Published:** 2025-12-13

**Authors:** James M. Dupree, Jane Kitaevich, Christina Agostino, Sitara Murali, Luca Borah, S. Kate Castle, Anna Kirkland

**Affiliations:** 1 Departments of Urology and Obstetrics and Gynecology, University of Michigan–Ann Arbor, Ann Arbor, USA; 2 Department of Political Science, University of Michigan–Ann Arbor, Ann Arbor, USA; 3 Department of Urology, University of Michigan–Ann Arbor, Ann Arbor, USA; 4 Department of Surgery, University of Michigan–Ann Arbor, Ann Arbor, USA; 5 Department of Sociology, University of Michigan–Ann Arbor, Ann Arbor, USA; 6 Kim Lane Scheppele Collegiate Professor of Women’s and Gender Studies, Arthur F. Thurnau Professor of Women’s and Gender Studies, Political Science, Sociology, and Health Management & Policy (by courtesy), University of Michigan–Ann Arbor, Ann Arbor, USA

**Keywords:** Infertility, In vitro fertilization, Health insurance, Legislation, State mandates, Policy

## Abstract

**Purpose:**

Sixty-five percent of US workers get their health insurance from self-insured employers who are exempt from state insurance coverage mandates. We evaluated if self-insured employers in states with in vitro fertilization coverage mandates offer insurance coverage for in vitro fertilization and other fertility treatments, and if so, we evaluated the details of that coverage.

**Methods:**

We qualitatively analyzed 165 health plan documents from 2019 to 2021 from 45 self-insured employers in seven states with in vitro fertilization coverage mandates (Arkansas, Connecticut, Illinois, Maryland, Massachusetts, New Jersey, New York).

**Results:**

A minority (41%) of self-insured employers in states with in vitro fertilization coverage mandates cover in vitro fertilization. Most health plans impose lifetime limits on in vitro fertilization use, with those limits split almost evenly between dollar-based and cycle-based limits. Finally, health plans in our sample from the finance and insurance, manufacturing, and educational services industries, and from non-union employers, had more comprehensive coverage for infertility testing and treatments.

**Conclusion:**

While state in vitro fertilization coverage mandates are important policy initiatives to improve access to in vitro fertilization, our findings suggest that state mandates are insufficient to expand access to all patients since a minority of self-insured employers in our sample covered in vitro fertilization. In addition, it is insufficient to label a health plan as simply “covering” or “not covering” in vitro fertilization; instead, the details of that coverage matter since the “coverage” may not be sufficient for the patient to afford even one in vitro fertilization attempt.

## Introduction

One in six couples in the United States (US) has infertility [1]. The World Health Organization (WHO) and the American Society for Reproductive Medicine (ASRM) both define infertility as a disease because it is a deviation in the function of the reproductive system [2, 3]. In the US, access to infertility care is largely mediated by health insurance coverage [4], since without health insurance coverage, a single in vitro fertilization (IVF) attempt can cost patients more than $18,000 [5]. Most reproductive-age adults receive health insurance from their employers, and employers provide health insurance for their employees in one of two ways: Employers can be fully insured or self-insured [6–8]. Fully insured employers buy health insurance products from insurance carriers (e.g., Aetna, Humana), and the insurance carriers bear the financial risk of insurance claims. Fully insured health plans are subject to state insurance mandates. Self-insured employers can design their own health insurance products, bear the financial risk of insurance claims themselves, and use an insurance carrier solely to administer their insurance plan. Self-insured health plans are exempt from state insurance mandates and instead are regulated under the federal Employee Retirement Income Security Act of 1974 (ERISA) [9]. Sixty-five percent of adults with employer-sponsored health insurance work for self-insured employers [6].

As of 2020, eleven states (Arkansas, Connecticut, Delaware, Hawaii, Illinois, Maryland, Massachusetts, New Hampshire, New Jersey, New York, Rhode Island) had attempted to improve access to IVF by passing laws mandating that fully insured employers in their states provide insurance coverage for IVF for their employees [10]. However, it is unknown how self-insured employers in these states choose to cover (or not cover) IVF for their employees. Self-insured employers in states with IVF coverage mandates may choose to provide insurance coverage for IVF so they can compete in the local labor market to recruit and retain workers. Alternatively, self-insured employers may choose not to include coverage for IVF in their health insurance plans due to concerns about cost or other factors [11].

It is difficult to study self-insured employers’ health plans because they are not required to file their health plan documents publicly, unlike fully insured health plans, federal and state employer plans, Medicare and Medicaid, and marketplace plans, all of which have health plan documents that can be found online. Surveys of employers about IVF coverage show increasing rates of coverage but do not examine health plan documents, lack information about coverage details, and do not categorize employers based on state or on self-insured vs. fully insured status [12]. Studies using insurance claims data only capture IVF usage, not coverage details [13]. Surveys of insurance carrier medical policies are revealing [14], but less relevant for self-insured employer coverage because the employer is the plan designer and the carrier simply administers the plan. Because of all of these research barriers, previous studies that examined employer-sponsored health insurance documents used small samples from single industries (e.g., 14 medical schools, 24 residency training programs) and did not specifically identify self-insured employers [15, 16]. Thus, these previous studies only examined IVF coverage for particular, smaller groups of employees.

Our goal was to evaluate if self-insured employers in states with IVF coverage mandates offered IVF coverage for their employees even when their state mandate does not require it, and if so, to analyze the details of those IVF coverage policies. For this research and to overcome the constraints of prior study designs, we established access to the largest known repository of insurance plan documents in the US. We performed a systematic content analysis of insurance plan documents from self-insured sponsors in states with IVF coverage mandates. Plan sponsors in our data are private employers, public employers, and labor unions. The term “sponsor” reflects this range of arrangements, but for simplicity, we will use the term employer in most of this paper. We hypothesized that self-insured employers in states with mandated IVF coverage would offer insurance coverage for IVF for their employees, even when they are not subject to their state’s mandate to cover it.

## Materials and methods

### Data source

Since there is no legal requirement for self-insured employers to file their health plan documents publicly, there is no pre-existing, public, comprehensive data source for self-insured employer health plan documents [17]. Health plan documents must be hand-gathered from internet sources in a laborious process. Thus, we established a data use agreement with Leverage Global Consulting that provided us access to their large, nationwide repository of employer-sponsored health insurance plan documents. Their proprietary database contained insurance plan documents from 2016 to 2021 for self-insured and fully insured employers. We have previously used the Leverage database to study insurance coverage for gender-affirming care with a different sample of documents [18, 19].

### Employer sample

Using the Leverage database, we identified health insurance plan documents from self-insured employers operating in seven (AR, CT, IL, MD, MA, NJ, NY) of the states with insurance mandates for IVF coverage as of 2020. The Leverage database identified which health plans were self-insured. We used the address listed on each employer’s website to determine where each employer was located. We categorized employers into industry types using the North American Industry Classification System (NAICS) [20].

### Document sample

We analyzed Summary Plan Description (SPD) and Summary of Benefits and Coverage (SBC) documents for employers in our sample. These documents are required by federal law to include details for employees about what is covered and not covered by their health insurance plans, and at what cost. But the law only requires that the documents are given to the beneficiary; they are not required to be publicly available. We excluded health plan documents from New York employers from 2016 to 2019 (*n* = 12) since the New York IVF coverage mandate became effective on January 1, 2020. There are no human subjects in this research, and no personally identifying information. This study was deemed exempt by the University of Michigan Institutional Review Board.

### Systematic content analysis and document coding

First, we created an a priori list of elements of insurance coverage that would be relevant for patients with infertility, including services covered (e.g., infertility diagnostic testing, treatment for underlying conditions, intrauterine insemination (IUI), IVF) and limits on coverage (e.g., dollar-based limits, IVF cycle number-based limits). This resulted in a list of 32 variables of interest. We then iteratively developed a coding protocol that was tested and revised by the research team.

Next, we used this coding protocol to perform a systematic content analysis of all Summary Plan Description documents to extract and code the relevant elements of infertility coverage [19]. All documents were independently analyzed and coded by two trained coders. Coders assessed coverage for each of the 32 variables of interest in each plan document at four levels of coverage: fully covered, partially covered, excluded, and not mentioned. We calculated inter-coder agreement before performing any adjudications using Cohen’s kappa coefficient. The Cohen’s kappa before any adjudications was 85%, indicating a high level of coding agreement between the two coders.

### Adjudication of discrepancies and missing data

We resolved disagreements between coders who entered opposite codes (“covered” and “excluded”) through adjudication of the code by a senior author (JMD or AK). We addressed missing entries in two ways. First, when the first coder entered “covered,” “partially covered,” or “excluded,” and the second coder did not enter a response, a senior author (AK) reviewed the missing values as the “second coder” and completed the missing entry. Second, when there was a first coder who entered “no mention” and a second coder who did not enter a response, we accepted “no mention” as the final value, assuming that the second coder left the entry blank because the variable was not mentioned.

### Descriptive analysis

We produced descriptive statistics to detail how many self-insured employers offer insurance coverage for different elements of infertility care, including diagnostic testing, treatment for underlying conditions causing infertility, fertility medications, IUI, IVF, and fertility preservation. Our descriptive statistics included measures of central tendency (e.g., the percent of health plans that provided coverage for IVF) and measures of variability (e.g., how coverage for IVF varies by employer factors such as industry).

## Results

We analyzed 165 health insurance plan documents from 45 self-insured employers in seven states (AR, CT, IL, MD, MA, NJ, NY). Table 1 depicts the number and percentages of health plans in our sample that covered one of thirteen key elements of infertility care, which broadly include evaluations, treatments, cryopreservation, and fertility preservation. Overall, 82% (*n* = 135) of health plans fully covered at least one element of infertility care. However, the spectrum of coverage varied widely. Fifty seven percent (*n* = 94) of health plans fully covered male and/or female diagnostic infertility evaluations, but 10 of those 94 plans (11%) offered no coverage for any additional types of infertility treatments. A minority (39%, *n* = 64) of plans fully covered treatment for underlying conditions. A majority (67%, *n* = 110) of health plans fully covered IUI, while less than half of the health plans (41%, *n* = 67) fully covered IVF and only 27% (*n* = 45) fully covered fertility medications.

**Table 1:** Percent of health plans (*n*) fully covering, offering partial coverage for, excluding coverage for, or not mentioning infertility services

	Fully covered	Partially covered	Excluded	Not mentioned
**Evaluation**				
Female evaluation	57% (94)	1% (2)	6% (9)	36% (60)
Male evaluation	49% (81)	4% (7)	5% (8)	42% (69)
Treatment of underlying conditions	39% (64)	6% (9)	10% (17)	46% (75)
Reversal of sterilization	3% (5)	1% (2)	89% (147)	7% (11)
**Treatment**				
IUI	67% (110)	2% (4)	24% (40)	7% (11)
IVF	41% (67)	9% (14)	44% (73)	7% (11)
Fertility medications	27% (45)	32% (52)	23% (38)	18% (30)
**Cryopreservation**				
Cryopreservation of eggs	2% (4)	10% (16)	67% (110)	21% (35)
Cryopreservation of sperm	2% (4)	10% (17)	64% (106)	23% (38)
Cryopreservation of embryos	6% (9)	13% (22)	53% (88)	28% (46)
**Fertility preservation**				
Oncologic fertility preservation	9% (14)	5% (8)	49% (80)	38% (63)
Iatrogenic fertility preservation	7% (11)	5% (8)	47% (77)	42% (69)
Planned fertility preservation	0% (0)	0% (0)	44% (73)	56% (92)

Cryopreservation of gametes and embryos is often necessary during IVF and can be costly since embryos may remain frozen for years, and storage can cost thousands of dollars [21, 22]. Table 1 depicts that cryopreservation costs were typically excluded by health plans in our sample, with only 2% (*n* = 4) of health plans fully covering the costs of egg cryopreservation, 2% (*n* = 4) fully covering the costs of sperm cryopreservation, and 6% (*n* = 9) fully covering the costs of embryo cryopreservation. We were also interested in insurance coverage for fertility preservation. Nine percent (*n* = 14) and 7% (*n* = 11) of health plans fully covered fertility preservation for oncologic and iatrogenic infertility, respectively. No health plans covered planned fertility preservation.

When a health plan covered IVF, the lifetime limits on that coverage varied widely. Table 2 depicts how many health plans contained limits on IVF care based on dollar or cycle amounts and the frequency and level of each type of limit. Overall, 50% (*n* = 83) of health plans specified a lifetime dollar- or cycle-based limit on IVF use. Thirty-two percent (*n* = 52) of plans included a lifetime dollar limit. Twenty-six percent (*n* = 42) of health plans included a lifetime cycle-based limit, and 7% (*n* = 11) of health plans included both dollar- and cycle-based lifetime limits on IVF use. Thirty-nine percent (*n* = 20) of the 52 health plans with a dollar-based lifetime coverage limit used a dollar amount between $15,000 and $20,000, which would cover about one IVF cycle. Twelve percent (*n* = 6) of the 52 health plans with a dollar-based coverage limit used a dollar amount ($5,000 to $10,000) that was less than the typical cost of one IVF cycle.

**Table 2:** Percent of health plans (*n*) with lifetime dollar or cycle-based limits on IVF use^a^

	Frequency
**Lifetime dollar cap range**	
$5,000–10,000	4% (*n* = 6)
$15,000–20,000	12% (*n* = 20)
$21,000–30,000	12% (*n* = 19)
$35,000–40,000	1% (*n* = 2)
$100,000	3% (*n* = 5)
Not mentioned	69% (*n* = 113)
**Lifetime cycle limit**	
1	7% (*n* = 13)
2	0% (*n* = 0)
3	9% (*n* = 15)
4	6% (*n* = 9)
5	0% (*n* = 0)
6	3% (*n* = 5)
Not mentioned	75% (*n* = 123)

^a^7% (11 plans) had both cycle and dollar limits

Finally, we were interested in differences in insurance coverage for infertility care based on employers’ industry and whether the plan was the result of labor union collective bargaining. Table 3 depicts the differences in coverage of infertility care by industry and union status. Health plans from the finance and insurance, manufacturing, and educational services industries had more prevalent coverage for infertility testing and treatments. Union plans were less generous than non-union plans on all dimensions of infertility coverage, with only 12% (*n* = 3) of union plans fully covering IVF as compared with 46% (*n* = 64) of non-union plans fully covering IVF.

**Table 3:** Percent of health plans (*n*) fully covering various infertility services, by industry type and union status

	Number of health plans	Evaluation for male infertility fully covered	Evaluation for female infertility fully covered	Treatment of underlying conditions fully covered	IUI fully covered	IVF fully covered	Medications fully covered
**Industry type**							
Healthcare	45	20% (9)	27% (12)	31% (14)	82% (37)	33% (15)	27% (12)
Finance and insurance	35	74% (26)	86% (30)	69% (24)	89% (31)	69% (24)	31% (11)
Manufacturing	22	86% (19)	86% (19)	55% (12)	77% (17)	59% (13)	41% (9)
Educational services	19	37% (7)	58% (11)	21% (4)	63% (12)	63% (12)	16% (3)
Building support management	10	29% (3)	36% (4)	0% (0)	0% (0)	0% (0)	0% (0)
Accommodation	11	58% (7)	58% (7)	17% (2)	17% (2)	17% (2)	17% (2)
Transportation and warehousing	6	33% (2)	33% (2)	33% (2)	0% (0)	0% (0)	17% (1)
Membership	6	100% (6)	100% (6)	67% (4)	100% (6)	0% (0)	100% (6)
Telecommunications	5	0% (0)	40% (2)	0% (0)	80% (4)	40% (2)	40% (2)
Retail trade	2	50% (1)	50% (1)	50% (1)	0% (0)	0% (0)	0% (0)
Arts and entertainment	2	100% (2)	100% (2)	100% (2)	100% (2)	0% (0)	0% (0)
Public administration	1	0% (0)	0% (0)	0% (0)	0% (0)	0% (0)	0% (0)
Construction	1	100% (1)	100% (1)	0% (0)	0% (0)	0% (0)	0% (0)
**Union status**							
Union plan	26	35% (9)	39% (11)	8% (2)	15% (4)	12% (3)	15% (4)
Non-union plan	139	52% (72)	60% (83)	45% (63)	76% (106)	46% (64)	30% (42)

## Discussion

Our study has three main findings. First, less than half of self-insured employers in states with IVF coverage mandates in our sample covered IVF. Second, about half of the health plans in our sample imposed lifetime limits on IVF use, with those limits split almost evenly between dollar-based and cycle-based limits. Third, health plans from the finance and insurance, manufacturing, and educational services industries and from non-union employers in our sample had more prevalent coverage for infertility testing and treatments. Overall, while state IVF coverage mandates are important policy initiatives to improve access to IVF, our findings suggest that state coverage mandates are insufficient to expand access to all patients since a minority of self-insured employers in our sample covered IVF.

We found that only 41% of self-insured employers in states with IVF coverage mandates provide full coverage for IVF for their employees. No broad, document-based studies of IVF coverage have been performed previously [15, 16, 23, 24]. The next-best available research about employer-sponsored insurance coverage for IVF comes from employer surveys, most of which were performed by the Mercer Corporation [11, 12, 23–25], and from small samples of medical schools or residency training programs [15]. None of these previous studies identified whether employers were self-insured or if the employers were in states with IVF coverage mandates. Mercer’s surveys found that the number of employers that offer IVF coverage has increased in recent years [23–25]. Prior to 2020, only about 25% of large companies (500 + employees) covered IVF [24]. But, in 2020, Mercer’s survey found that 27% of large employers reported covering IVF, and this coverage rate increased to 36% in 2021 [23, 25]. Our document-based finding that 41% of self-insured health plans in states with IVF mandates offer IVF coverage aligns with Mercer’s 2021 survey findings and adds important new data since all employers in our sample were in states with IVF coverage mandates. Our findings suggest that state IVF coverage mandates are not associated with self-insured employers in those states covering IVF at a much higher rate than found in recent nationwide surveys of large employers.

Union membership can help employees gain health insurance coverage, but the 26 union plans in our sample were markedly less generous for infertility benefits [26]. Only 3 of 26 union plans covered IVF (12%) compared to 46% of non-union plans covering IVF. (The overall 41% coverage rate combines all plans.) Union plans in our sample included those from traditionally male-dominated unions for the trades, but also those for hotel workers, service workers, and teachers. Our findings suggest that the health insurance coverage advantage that union membership confers does not yet extend fully to fertility benefits.

We also found considerable variability in the specifics of the infertility coverage offered by these different health plans, especially for lifetime limits on IVF use. Several health plans placed dollar- or cycle-based lifetime limits on IVF use, and in some cases, the dollar-based limit was less than the cost of a typical IVF cycle. There has been little research about IVF limits in health plans. The employer surveys conducted by Mercer did not ask about lifetime or dollar limits on IVF coverage [23–25]. The previous studies that examined health plan documents from 14 medical schools or 24 US or Canadian residency training programs found a wide range of coverage limits from $10,000 to $100,000 and $7,500 to $25,000, respectively [15, 16]. Based on our findings, we conclude that it is insufficient to label a health plan as simply “covering” or “not covering” IVF; instead, the details of that coverage matter since the “coverage” may not be enough to help the patient afford even one IVF attempt.

Our study has several limitations and strengths. First, we are limited to the selection of health plans available in the Leverage database at the time we performed our study. Because there is no other database that captures the universe of private, employer-sponsored health plans, it is difficult to estimate how representative our sample is. Nevertheless, our study draws on a novel data source and performs the largest known systematic content analysis of health insurance plan documents for infertility care to date. Second, our sample is geographically limited to the subset of seven states with IVF coverage mandates that were found in the Leverage database in 2021. However, these seven states are several of the largest states in the country and collectively represent 19% of the US population [27]. While Mercer’s surveys can be used as benchmarks for national coverage rates (subject to the limitations mentioned above), Mercer does not separate their findings by state. Finally, we did not perform an analysis of the insurance plans that insurance carriers sell to fully insured employers in these states with fertility mandates. We did not analyze these fully insured plans because we assumed those plans conform to state laws requiring them to offer insurance coverage for IVF. Our study also has several strengths that overcome the constraints of prior study designs, including our original data, direct access to health plan documents, extensive coding from both legal and medical perspectives, and its focus on employers operating in states with IVF coverage mandates but exempt from those mandates because of their self-insured status.

Our findings have important implications for patients, employers, advocates, and policymakers. For patients, these findings highlight the importance of reading the specific coverage details found within their health plan documents. Even if their employer states that IVF is “covered,” the details of that coverage may limit employees’ access to certain infertility treatments. Parsing these documents can be challenging for patients, and our findings suggest that more clarity and standardization are needed for transparency. For employers, our findings offer important benchmarks for what other health plans in their industry or state may offer. It is notable that even when employees collectively bargain as part of a union, benefits tend to be less generous in infertility care compared to the other plans in our study. For advocates and policymakers, our findings emphasize that while state IVF coverage mandates are important ways to improve access to IVF care for many patients, they are insufficient to expand access to all patients. Since 65% of adults with employer-sponsored insurance work for self-insured employers, there are many employees who are not reached by state IVF coverage mandates.

## Data Availability

No datasets were generated or analyzed during the current study.
